# Southern Dental Association

**Published:** 1891-07

**Authors:** 


### SOUTHERN DENTAL ASSOCIATION.

This energetic organization, which represents the profession in the South, will meet this year at Morehead City, N. C., commencing August 11, 1891. The announcement did not reach us in time to notice in our last number.

We desire also to modify the statement in the same number in our article on "Conventions," in which the inference was drawn that the Southern was arranged with "permanent members" and "delegates," as the American Dental Association. This we learn is a mistake, they having but two classes,—"Active and Honorary."

